# Phylogeographic reconstruction of a bacterial species with high levels of lateral gene transfer

**DOI:** 10.1186/1741-7007-7-78

**Published:** 2009-11-18

**Authors:** Talima Pearson, Philip Giffard, Stephen Beckstrom-Sternberg, Raymond Auerbach, Heidie Hornstra, Apichai Tuanyok, Erin P Price, Mindy B Glass, Benjamin Leadem, James S Beckstrom-Sternberg, Gerard J Allan, Jeffrey T Foster, David M Wagner, Richard T Okinaka, Siew Hoon Sim, Ofori Pearson, Zaining Wu, Jean Chang, Rajinder Kaul, Alex R Hoffmaster, Thomas S Brettin, Richard A Robison, Mark Mayo, Jay E Gee, Patrick Tan, Bart J Currie, Paul Keim

**Affiliations:** 1 Center for Microbial Genetics and Genomics, Northern Arizona University, Flagstaff, Arizona, USA; 2 Institute of Health and Biomedical Innovation, Queensland University of Technology, Kelvin Grove, Australia; 3 Menzies School of Health Research, Charles Darwin University, Darwin, Australia; 4 Pathogen Genomics Division, Translational Genomics Research Institute, Phoenix, Arizona, USA; 5 Bacterial Zoonoses Branch, Division of Foodborne, Bacterial, and Mycotic Diseases, National Center for Zoonotic, Vector-Borne, and Enteric Diseases, Centers for Disease Control and Prevention, Atlanta, GA, USA; 6 Northern Arizona University, Department of Biological Sciences, Environmental Genetics & Genomics Facility, Flagstaff, Arizona, USA; 7 Biosciences, Los Alamos National Laboratory, Los Alamos, New Mexico, USA; 8 Defense Medical and Environmental Research Institute, Singapore, Republic of Singapore; 9 US Geological Survey, Denver Federal Center, MS 939 Denver, Colorado, USA; 10 University of Washington Genome Center and Division of Medical Genetics, Department of Medicine, University of Washington, Seattle, Washington, USA; 11 DOE Joint Genome Institute, Bioscience Division, Los Alamos National Laboratory, Los Alamos, NM, USA; 12 Department of Microbiology & Molecular Biology, Brigham Young University, Provo, UT, USA; 13 Genome Institute of Singapore, Singapore, Republic of Singapore; 14 Northern Territory Clinical School, Royal Darwin Hospital, Darwin, Australia; 15 Program in Computational Biology and Bioinformatics, Yale University, New Haven, Connecticut, USA

## Abstract

**Background:**

Phylogeographic reconstruction of some bacterial populations is hindered by low diversity coupled with high levels of lateral gene transfer. A comparison of recombination levels and diversity at seven housekeeping genes for eleven bacterial species, most of which are commonly cited as having high levels of lateral gene transfer shows that the relative contributions of homologous recombination versus mutation for *Burkholderia pseudomallei *is over two times higher than for *Streptococcus pneumoniae *and is thus the highest value yet reported in bacteria. Despite the potential for homologous recombination to increase diversity, *B. pseudomallei *exhibits a relative lack of diversity at these loci. In these situations, whole genome genotyping of orthologous shared single nucleotide polymorphism loci, discovered using next generation sequencing technologies, can provide very large data sets capable of estimating core phylogenetic relationships. We compared and searched 43 whole genome sequences of *B. pseudomallei *and its closest relatives for single nucleotide polymorphisms in orthologous shared regions to use in phylogenetic reconstruction.

**Results:**

Bayesian phylogenetic analyses of >14,000 single nucleotide polymorphisms yielded completely resolved trees for these 43 strains with high levels of statistical support. These results enable a better understanding of a separate analysis of population differentiation among >1,700 *B. pseudomallei *isolates as defined by sequence data from seven housekeeping genes. We analyzed this larger data set for population structure and allele sharing that can be attributed to lateral gene transfer. Our results suggest that despite an almost panmictic population, we can detect two distinct populations of *B. pseudomallei *that conform to biogeographic patterns found in many plant and animal species. That is, separation along Wallace's Line, a biogeographic boundary between Southeast Asia and Australia.

**Conclusion:**

We describe an Australian origin for *B. pseudomallei*, characterized by a single introduction event into Southeast Asia during a recent glacial period, and variable levels of lateral gene transfer within populations. These patterns provide insights into mechanisms of genetic diversification in *B. pseudomallei *and its closest relatives, and provide a framework for integrating the traditionally separate fields of population genetics and phylogenetics for other bacterial species with high levels of lateral gene transfer.

## Background

Efforts to understand the evolutionary history of organisms have benefited from the availability of increasing amounts of molecular data, especially whole genome sequences (WGSs). The availability of multiple WGSs has led to more accurate reconstructions of phylogenetic relationships within several bacterial species [1-9], but all of these studies have been limited by a small number of WGSs (19 or fewer genomes). The availability of multiple WGSs per species is currently quite rare, but the cost of generating WGSs continues to decline and it is anticipated that future phylogenetic studies will routinely employ multiple WGSs.

Due to their short evolutionary history and clonality, *Bacillus anthracis *[5] and *Mycobacterium tuberculosis *[10] were ideal models for pioneering phylogenetic work using multiple WGSs, but hurdles in phylogenetic reconstruction persist for other species. The genomes of these two species exhibit almost no homoplasy (the appearance of similar character states in unrelated samples due to evolutionary convergence or parallelisms) due to their recent species derivation and complete clonality. Thus, character differences, as measured by single nucleotide polymorphisms (SNPs), are assumed to have arisen only once in their evolutionary history. Also, these two species exhibit no evidence of conspecific lateral gene transfer (LGT), which can cause *apparent homoplasy *by placing alleles with common origins in different genetic backgrounds. In contrast, most bacterial species, including *Burkholderia pseudomallei*, have a longer history of mutation accumulation, as well as a history of LGT [11-13], which increase the probability of homoplasy and *apparent homoplasy*, respectively. Thus, for all but the most recently emerged and clonal species, fine-scale phylogenetic reconstruction has been elusive using common genetic markers. Recent sequencing efforts for *B. pseudomallei *and other closely related species provided the opportunity for pioneering phylogenetic work on a species with high levels of LGT.

*B. pseudomallei *causes the severe disease melioidosis [14] and is widely distributed in soil and fresh water in Southeast Asia and tropical Australia [15]. Animal to animal transmission is rare but a wide variety of animals can be infected [16,17], reseeding nearby areas [17,18] and providing limited dispersion for this otherwise immobile species. These small-scale movements should be reflected in the population structure of *B. pseudomallei*, with geographic barriers such as oceans being traversed rarely or not at all. A monophyletic group of isolates within the *B. pseudomallei *group has diverged to become an equine pathogen, *B. mallei *[16], which does not survive well in soil. Like *B. pseudomallei*, the closely related *B. thailandensis *and *B. oklahomensis *live in soil but are much less pathogenic and are phylogenetically distinct from *B. pseudomallei*/*B. mallei *[19].

Various molecular methods have been used for phylogenetic reconstruction of these *Burkholderia *species, with different levels of success. Multiple-locus variable number tandem repeat (VNTR) analysis (MLVA) of *B. pseudomallei *and *B. mallei *is effective for determining relationships among very closely related isolates, but not broad patterns of relatedness [20,21]. Multilocus sequence typing (MLST) of seven housekeeping genes [22] can be used to identify epidemiologically linked isolates of the same sequence type (ST) and determine phylogenetic relationships at a species level [16], but efforts to infer relationships among STs within *B. pseudomallei *have yielded little statistical support [16,23,24]. This is due to homologous recombination within and possibly among *B. thailandensis*, *B. pseudomallei*, and *B. mallei *[11-13,24,25], as well as limitations of restricted gene sampling in highly recombining populations [26]. Microarray-based comparative gene hybridization analysis of 23 strains from these three *Burkholderia *species avoided problems associated with limited gene sampling by targeting close to 7,000 open reading frames discovered from the WGS of a single *B. pseudomallei *isolate, K96243 [27]. However, the subsequent phylogeny derived from this work was heavily weighted towards isolates from Southeast Asia [27] and may not be representative of the global evolution of these species. Thus, the need for a comprehensive phylogeny with extensive character sampling persists.

Whole genome SNP phylogenies are highly accurate in terms of defining both branching order and branch lengths, despite collapsed secondary branches that lead to isolates that have not been sequenced [5,28,29]. SNPs are more evolutionarily informative than most other types of molecular markers due to intrinsically slow mutation rates, few character states, and extensive distribution across the entire genome. In addition, a large number of shared, orthologous SNP loci facilitate robust characterization of deep relationships and high resolution among closely related individuals [28,30,31]. However, because SNPs are relatively rare and scattered throughout a genome, WGSs from multiple strains are required for identification.

Here, we construct a robust, large-scale phylogeny of *B. pseudomallei *and its close relatives, overcoming significant problems associated with LGT and SNP discovery by using a large number of orthologous SNP loci distributed across the entire genome and shared among 43 fully sequenced genomes. We compare these findings to broad population patterns determined using sequence data from seven housekeeping genes in a global collection of >1,700 isolates. To our knowledge, this broad-scale integration of phylogenetics from whole genome sequence comparisons and population genetics from extensive MLST data in a spatial context is unprecedented for this species and provides a model for assessing the dispersal and differentiation of other bacteria that have high levels of recombination.

## Results and Discussion

### Phylogenetic patterns revealed by shared, orthologous SNPs derived from WGSs

**Figure 1:**
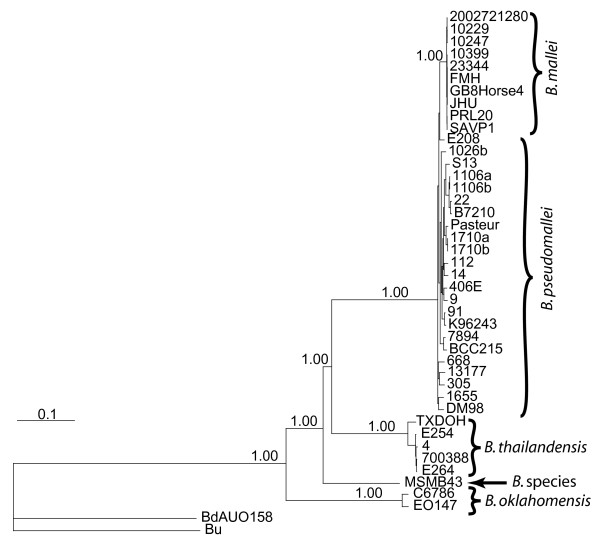
**Bayesian phylogenetic analysis of 11,208 single nucleotide polymorphisms shared among 43 whole genome sequences from six species**. Credibility values for major clades are included. *B. dolosa *and *B. ubonensis *were used to root this tree.

Bayesian phylogenetic analysis of 11,208 orthologous SNPs shared among all WGSs confirmed the distant relationship of *B. thailandensis *and the monophyletic derivation of *B. mallei *from a *B. pseudomallei *lineage (Figure 1). These SNPs were identified by comparing and searching WGSs of 23 *B. pseudomallei*, ten *B. mallei*, five *B. thailandensis*, and five other near neighbor isolates (Additional file 1), followed by removal of paralogous and non-shared SNP loci. The phylogenetic position of MSMB43, an Australian isolate with many *B. thailandensis *characteristics, appears sister to *B. thailandensis *and *B. pseudomallei/mallei*, a result consistent with MLST phylogenetic analysis [32].

**Figure 2:**
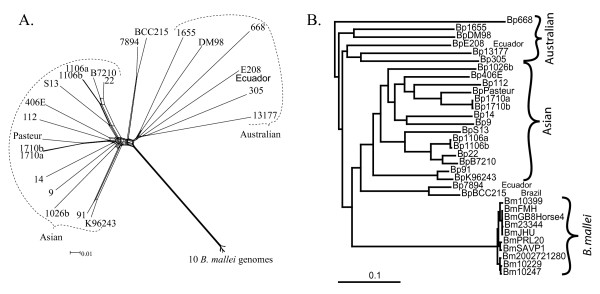
**Phylogenetic analysis of 14,544 single nucleotide polymorphisms shared among 33 whole genome sequences from two species**. Splits decomposition analysis provides a visual account of character state conflict within the dataset **(A)**. Bayesian phylogenetic analysis of these SNPs result in clade credibility values of 1.00 for all bifurcations (except 23344/JHU = 0.96) **(B)**. *B. thailandensis *was used to root this tree (see Additional file 3).

Excluding distant taxa increases the proportion of the genome that is shared among remaining taxa. Thus, when progressively smaller groups of isolates are compared, a larger number of orthologous shared SNP loci can be found. Excluding distant taxa also improves phylogenetic accuracy by avoiding problems associated with long branch attraction [33]. This approach, which provided high levels of statistical support, utilized results from previous, more global analyses to identify outgroups for rooting phylogenetic trees. In this way we were able to confirm the location of MSMB43 by eliminating the WGSs of *B. ubonensis *and *B. dolosa *from our SNP discovery procedure and subsequent phylogenetic analysis (tree not shown). Similarly, by retaining only *B. thailandensis *as an outgroup for rooting, we searched for the first isolate or group of isolates to diverge from the *B. pseudomallei/B. mallei *clade. The close proximity of the deep bifurcation points in this clade is likely due to a radiation event, or to the cohesive forces of LGT that led to a limited number of SNPs separating these bifurcation points. This resulted in unresolved deep branches despite using 17,718 SNPs in a Bayesian analysis (Additional file 2). We further analyzed these data using Maximum Likelihood, Maximum Parsimony, and Neighbor-Joining phylogenetic methods alongside a data set of 67,644 SNPs obtained by relaxing the criteria for SNP selection (see methods and Additional file 3). All analyses including the Bayesian analysis show that the Australian isolates share a more ancient common ancestor than the Asian isolates, and both Maximum Parsimony and Neighbor-Joining analyses suggest that *B. pseudomallei *isolate 668 was the first isolate to diverge from the *B. pseudomallei/B. mallei *clade. Thus, we used *B. pseudomallei *isolate 668 as an outgroup for rooting our *B. pseudomallei/B. mallei *phylogeny, which was constructed using 14,544 SNPs shared only among *B. pseudomallei *and *B. mallei *genomes (Figure 2). The conclusions that we draw are contingent on an Australian root to this tree and not isolate 668 in particular.

All *B. pseudomallei *bifurcations received the highest possible level of statistical support (Figure 2). The Australian population of *B. pseudomallei *is more ancient than either the Asian *B. pseudomallei *group or the *B. mallei *group. The Australian isolates form a deep paraphyletic group within the *B. pseudomallei/B. mallei *phylogeny, and the most divergent isolates in this group are five isolates from Australia and E208 from Ecuador (Figure 2). The remaining isolates form two clades: the *B. mallei *clade and a group of *B. pseudomallei *dominated by Asian isolates. The phylogenetic position of *B. mallei *confirms previous research suggesting that this species arose from a *B. pseudomallei *lineage and experienced a recent radiation [16]. Our results suggest that none of the *B. pseudomallei *isolates included in this study are closely related to the *B. mallei *clone, and all Asian *B. pseudomallei *isolates fall into a single coherent group that diverged relatively recently, after the split with *B. mallei*.

LGT events may have had only a slight effect on phylogenetic topology, suggesting that while most events are spread evenly throughout the *B. pseudomallei *phylogenetic space, some *highways *of gene sharing may exist. Homoplastic SNPs are located throughout shared genomic regions and are interspersed with non-homoplastic SNPs, suggesting that all regions may have been subject to LGT events and that such events likely involved small stretches of DNA (Additional file 4a). Our test of residual homoplasies (materials and methods) resulted in a tree that is remarkably similar to the previous topology (Additional file 4b,c). Topological similarities have three explanations: 1) a lack of preferential transfer of DNA always involving the same lineages (no *highways *of gene sharing), 2) convergent or reverse mutations evenly spread throughout the phylogeny, and 3) sequencing errors evenly spread throughout phylogenetic space. Stochastic variation in homoplastic mutations and sequencing errors may explain topological differences within the *B. mallei *clade, which was mostly supported by non-homoplastic characters. Stochastic variation in all three parameters may be sufficient to cause topological differences among *B. pseudomallei *relationships, however as three of the four changed bifurcations remain statistically robust, we suspect that some LGT events consistently involved the same lineages. The congruency between trees created with homoplastic SNPs and all orthologous shared SNPs further increases our confidence that depicted phylogenetic relationships provide a reasonable hypothesis for the actual patterns of descent for the individual isolates. As the addition of more taxa increases phylogenetic accuracy [34], we suspect that the actual patterns of vertical descent will become even more distinct as more genomes are sequenced. This confirms the appropriateness of depicting the core evolutionary history of this set of organisms with a phylogenetic tree, rather than a network, as individual LGT events do not involve a large enough portion of the genome to disrupt the core phylogenetic patterns. In other words, despite high levels of LGT, the underlying core evolutionary trajectory can be determined and follows a bifurcating pattern. Indeed, as the evolutionary history of individual genes are determined, mapping these events onto the core phylogenetic tree will provide insights into gene flow within a species and will cause the core tree to appear more network-like. These analyses reveal the value and necessity of whole genome orthologous SNPs for defining patterns of descent even in organisms that are not completely clonal, with high levels of LGT. Sequence comparisons from a small number of genes such as MLST schemes would be grossly insufficient for defining these relationships.

### Gene flow dynamics revealed by MLST data

While the use of 23 WGSs from a single species is a high number for microbial phylogenetic studies, this number will seem more and more diminutive as the cost and speed of sequencing technologies increase. However, until the day when hundreds of genomes from a single species are sequenced, phylogenetic analyses will suffer from limited taxon sampling that may not represent natural diversity. It is therefore imperative that such phylogenetic information be integrated with data from a wider sampling of isolates, even though genotyping data will be more limited. Despite the limitations of MLST and the program eBURST [35] for phylogenetic inferences to determine exact evolutionary relationships between individual isolates [26,36], a large amount of MLST data nonetheless represents a valuable resource from which population-level trends can be gleaned. Analysis of allele frequencies can facilitate recognition of distinct populations, and comparisons of allelic diversity among populations are informative since ancient populations are expected to be more diverse than more recent populations, wherein genetic diversity is limited by founder effects [37]. Also, levels of intra- and inter-population connectedness, as measured by allele sharing, can be suggestive of levels of horizontal gene transfer and relatedness. Lastly, MLST data can be used to estimate levels of recombination that provide insights into LGT frequencies from measuring homoplasy, the standardized index of association [38] and the relative contribution of recombination and mutation in generating diversity [39]. We therefore analyzed MLST data from >1,700 isolates of *B. thailandensis, B. pseudomallei*, and *B. mallei *from an online database http://bpseudomallei.mlst.net downloaded on July 28, 2008. *B. pseudomallei *isolates in this database were collected from human and animal infections as well as a variety of environmental sources. As might be expected, STs that are found in clinical and animal cases are a subset of those found in the environment. Approximately 47% of STs are from Southeast Asia, 45% are from Australasia, and 8% are from other geographic regions.

**Figure 3:**
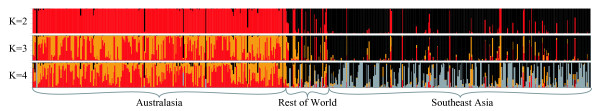
**Estimated population structure of *B. pseudomallei*/*mallei *using multilocus sequence typing data**. Each thin vertical line represents an ST and is divided into K portions that represent the estimated membership in K populations. Geographic affiliations of STs are labeled below the figure.

The extant *B. pseudomallei *form two populations: one composed largely of Australian isolates and one composed largely of Southeast Asian isolates (Φ_PT _= 0.117; *P *= 0.001; Figure 3). The existence of these distinct populations was confirmed using a systematic analysis of MLST data. A Bayesian-based clustering algorithm [40] was used to assign individual STs to populations with no *a priori *determination of the groupings (Figure 3). For each iteration, each ST is assigned to one of *K *populations. The proportion of iterations where a ST is assigned to each population is indicated by different colors and sums to one. Sorting STs into three geographically based categories (Australasia, Southeast Asia, and the rest of the world) confirmed the existence of two major populations and their geographic relationship. Increasing the value of *K *retained the two major populations but with further subdivision in both, indicating that the coherence of either population is not merely an artefact of intensive sampling in a single geographic region. STs incorrectly attributed to a geographic region either by mistake or by not taking into account the travel history of a patient [41] are easily identified by colored bars that are distinct from the surrounding vertical bars, indicating that they are genetically more similar to a different group, and providing further confidence in these results. STs from *the rest of the world *did not form a separate population even as *K *was increased, but rather were assigned to either the Australasian or Southeast Asian population, again suggesting that the observed patterns are not the result of uneven geographic sampling. Most of these STs are from isolates found in temperate geographic regions not normally associated with *B. pseudomallei*. It seems likely that such isolates are associated with either recent travel to endemic areas, or relatively recent introduction events. The statistically significant Φ_PT _value suggests that these two populations are sexually isolated from each other, while F_ST _values show the divergence of each population from an estimated ancestral population (F_ST _= 0.03 and 0.21 for the Australasian and Southeast Asian populations, respectively). The low divergence of the Australasian population and high divergence of the Southeast Asian population is expected, given our phylogenetic analyses which show that the Australasian population is paraphyletic and ancestral to the monophyletic Southeast Asian population.

**Figure 4:**
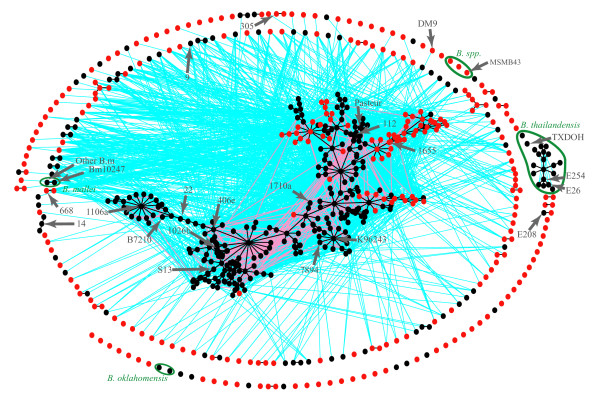
**eBURST population snapshot of *B. pseudomallei *and closely related species**. Dots represent individual MLST sequence types from Australia (red) and other countries (black). Single locus variants are connected by black (to predicted clonal complex founder) and pink lines (alternative single locus variants), whereas double locus variants are connected by blue lines. Isolates with WGSs are indicated, except BCC215, 91, 4, and 700388 as their sequence types are novel and are not yet included in the database.

The ancestral nature of the Australasian *B. pseudomallei *population was also confirmed with other analyses. An eBURST population snapshot of the MLST data depicts the connectedness of all 641 STs identified among the >1,700 isolates (Figure 4). The periphery of this population snapshot is dominated by singleton STs, mostly from Australia, that share few, if any, loci with other STs. The large central complex contains STs that share many alleles across the seven loci; these isolates are mostly from Southeast Asia, although some are Australasian. The next largest complex is comprised solely of *B. thailandensis *STs, none of which share >4 alleles with any STs from the other species. Conversely, *B. mallei *is represented by just two STs that differ from each other by a single SNP in one locus, and also share as many as five loci with some *B. pseudomallei *STs.

**Figure 5:**
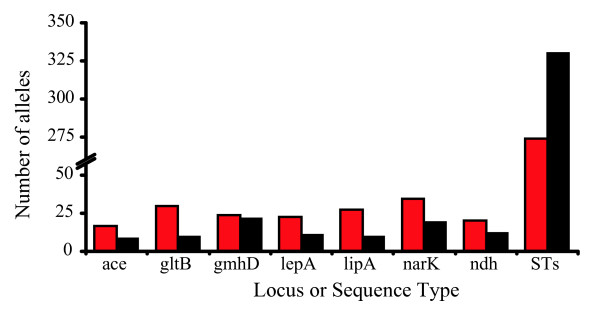
***B. pseudomallei *multilocus sequence typing diversity of isolates from Australia, Papua New Guinea, and New Caledonia (red), and the rest of the world (black)**. Allelic diversity at each locus is greater for the 811 isolates from Australia, Papua New Guinea, and New Caledonia than the 801 isolates from the rest of the world, suggesting an ancestral Australasian population. Conversely, the number of sequence types (STs) found in the rest of the world is greater than the number found in Australasia, suggesting lower levels of recombination in Australasia.

**Figure 6:**
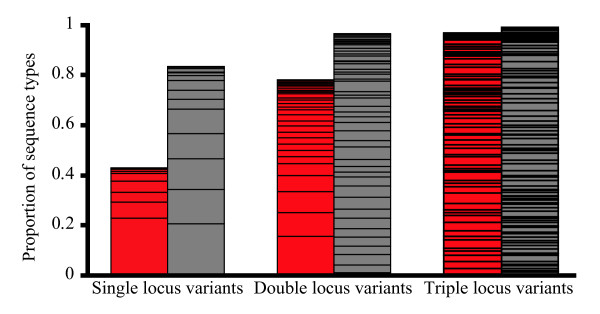
**Depiction of higher interconnectivity of Southeast Asian sequence types compared to Australian sequence types**. Red bars represent Australian STs and gray bars represent Southeast Asian STs. Height of each bar is the proportion of STs that possess at least one single, double, or triple locus variant. Horizontal lines represent unit increments of the numbers of variants for each category (*e.g.*, 0.23 of the Australian STs have only one single locus variant, whereas 0.06 have two single locus variants and 0.04 have three single locus variants). Sum of columns for each region is greater than one as each ST can have variants in each column.

There is a high degree of interconnectedness in the Southeast Asian population of *B. pseudomallei *that is not observed in the Australasian population (Figure 4). The Southeast Asian population contains a smaller number of MLST alleles, but they are mixed into a greater number of STs compared to the Australasian isolates (Figure 5). While the lower allelic diversity in the Southeast Asian population may be due to purging genetic novelties through recombination, this pattern is consistent with founder effects. Indeed, the greater allelic diversity of the Australasian population and its greater SNP-based diversity are consistent with the ancestral nature of the Australasian population as defined by phylogenetic analysis of the WGSs. The greater number of single, double, and triple locus variants in the Southeast Asian population suggests that this population recombines more frequently than the Australasian population (Figure 6), or the Australasian population contains a much deeper gene pool, resulting in any two isolates having a lower probability of obtaining the same MLST allele.

**Figure 7:**
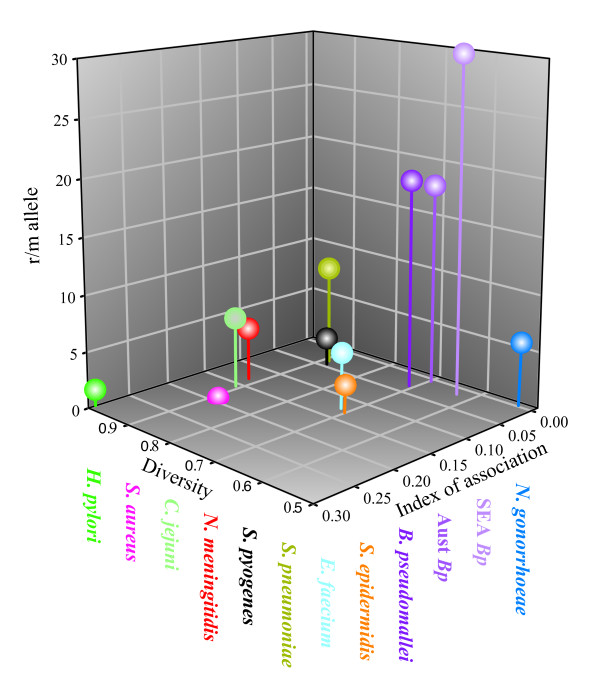
**Comparison of population metrics across 11 bacterial species**. The per-allele recombination to mutation parameter (r/m allele) suggests that *B. pseudomallei *alleles are between 18 and 30 times more likely to change by recombination rather than mutation. This value is higher than for any other bacterial species yet reported. The low standardized index of association suggests that these populations approach panmixia, and Nei's diversity index suggest that these species are less diverse than many other species.

Other measurements of the extent of LGT within a species suggests that recombination plays a much greater role than mutation in generating diversity in MLST loci for *B. pseudomallei*. We measured the standardized index of association [38], the relative contribution of recombination and mutation in generating diversity [39], and the average Nei's [42] diversity across MLST loci, for *B. pseudomallei *as well as 10 other species, some of which are often cited as being highly recombinagenic with regard to the relative contribution of recombination versus mutation. For *Neisseria meningitidis *[43], *Streptococcus pneumoniae *[43], *Enterococcus faecium *[44], *Staphylococcus epidermidis *[45] and *Staphylococcus aureus *[46], we used previously published values for the ratio of recombination to mutation. All other calculations (excluding those for *B. pseudomallei*) were based on MLST data from online databases (http://www.mlst.net and http://pubmlst.org, accessed May 23, 2009) (Figure 7 and Additional file 5). The recombination to mutation ratio is indicative of the contribution of recombination relative to mutation in generating allelic diversity [39] which can be measured by Nei's diversity index [42]. The index of association (I_a_^s^) measures the degree of association between loci where low values are indicative of linkage equilibrium and high levels of LGT [47]. Higher I_a_^s ^values do not necessarily indicate clonality as linkage disequilibrium can be caused by population structuring of a highly recombinant species [47]. For *H. pylori*, recombination gives rise to new MLST alleles only 1.5 times as often as mutation; therefore, the extremely high diversity can only be the result of high rates of both mutation and recombination. Given such high rates of recombination, the high index of association must be indicative of highly structured populations as has previously been reported [37]. In contrast to *H. pylori*, recombination in *B. pseudomallei *is between 18 and 30 times more likely to generate new alleles than mutation, a result consistent with previous analyses [48]. The low diversity in *B. pseudomallei *suggests that neither recombination nor mutation are very frequent while low index of association values suggest that this population is almost panmictic although significant linkage disequilibrium exists (*P *< 0.05). The overall population cannot be completely panmictic as population genetic analyses show two distinct populations. Thus panmixia must occur within populations. As might be expected by the greater interconnection of Southeast Asian STs compared to Australian STs (Figures 4 and 6), the recombination to mutation ratio for the Southeast Asian population is approximately 1.7 times higher than in the Australian population.

## Conclusion

The approaches described here provide a framework for phylogenetic analysis of species such as *B. pseudomallei *with high levels of LGT and homoplasy. Selecting only SNPs from whole genome comparisons eliminated faster evolving loci that are more prone to homoplasy [49], and sampling >14,000 SNPs spread across the genome reduced the confounding effects of convergent evolution and LGT. Deleted and duplicated genomic regions in *Burkholderia *are frequent [11,21,50,13] and can lead to missing data and sampling paralogous rather than orthologous loci [51,52], respectively. We therefore selected loci that were always present but not duplicated in any of the sequenced genomes. High clade credibility values coupled with a non-conflicting phylogenetic pattern of homoplastic SNPs provided confidence in the phylogenetic hypotheses presented here. The inferred phylogenies are a meaningful approximation of descent, and not simply a depiction of the inevitable stochastic variation in similarity that would be present in products of any finite random sampling of an infinitely panmictic population.

The validity of using phylogenetic trees to depict the evolutionary history of organisms exhibiting LGT has been hotly debated, with some authors championing web-like structures to depict instances of reticulate evolution [53] and others suggesting the importance and appropriateness of discerning patterns of vertical inheritance [54]. Certainly, intra- and interspecific genetic exchange has shaped the genome of extant *Burkholderia *isolates. However, although a large proportion of the genome may have been shaped this way over evolutionary time, only a very small portion of the genome is laterally inherited from generation to generation. Thus, a phylogenetic tree remains a valid way of representing the major patterns of descent for these species. On such a tree, the small connective threads that depict LGT and discordant individual gene phylogenies can subsequently be strung as individual genes are studied.

It is likely that the most recent common ancestor to *B. pseudomallei *existed on the Australian continent. Our phylogenetic analyses indicate a tendency for Australian *B. pseudomallei *isolates to be associated with a more ancient common ancestor compared to other isolates. This pattern also is supported by completely independent MLST results from 599 *B. pseudomallei *STs that showed that the Australasian population is defined by greater allelic diversity and fewer shared alleles. The presence of *B. thailandensis *isolates in Australia and the phylogenetic position of *Burkholderia *sp. MSMB43 point to the possibility that *Burkholderia *sp. MSMB43, *B. thailandensis, B. pseudomallei*, and *B. mallei *isolates are all descendants from an Australian *B. thailandensis*-like isolate, although this pattern is based on very few *B. thailandensis *and *Burkholderia*. sp. isolates. As more *B. thailandensis *isolates are discovered, their phylogenetic and geographic associations will be critical for confirming or rejecting this provisional hypothesis.

The monophyletic *B. mallei *clade diverged from *B. pseudomallei *before the current Southeast Asian population was established (Figure 2). The long branch leading to *B. mallei *strains suggests a long passage of time before a rapid radiation led to the extant population. A high consistency index among SNPs from whole genome comparisons of *B. mallei *strains provides evidence for a completely clonal mode of descent for this species since its relatively recent radiation, in contrast with *B. pseudomallei*. The lack of LGT among *B. mallei *isolates is not surprising given the loss of recombination opportunities associated with host sequestration and inability to thrive in the environment; it is likely that LGT between *B. mallei *and *B. pseudomallei *has not occurred for these same reasons. Although host specialization may account for the differential rates of LGT between the *B. pseudomallei *and *B. mallei *populations, other barriers may influence LGT among *B. pseudomallei *populations.

The mechanistic basis for high recombination frequencies observed in Southeast Asian populations of *B. pseudomallei*, compared to Australian populations, is of considerable interest. As sequences diverge, the likelihood of homologous recombination decreases [55-58]. Therefore, perhaps the greater genetic distances among Australian *B. pseudomallei *strains may, in part, explain lower levels of LGT in this population versus the more closely related and more connected Southeast Asian population. However, *B. thailandensis *shares more alleles with the Southeast Asian population of *B. pseudomallei *than with the Australian population (7:1), providing some evidence that LGT between species does occur despite genetic divergence. Different levels of LGT among populations may be due to greater abundance of *B. thailandensis *in Southeast Asia, providing greater opportunities for physical contact and LGT. In Australia, the typically lower abundance of *B. pseudomallei *in the environment [59] may account for lower rates of LGT in comparison to the Southeast Asian population [60]. Large, intensively farmed artificial wetlands such as the rice paddy fields of Thailand may favor high cell densities and mobility of strains. Conversely, the largely tropical savannah areas of Northern Australia dispersed over vast distances with limited low density grazing and human populations would be expected to impede gene flow [61]. A third scenario is that these populations may have evolved differential intrinsic LGT rates, however we have no evidence to support this hypothesis.

*B. pseudomallei *is subdivided into two distinct subpopulations with distinct geographic distributions that are separated by Wallace's Line. For hundreds of years naturalists have noted a tendency for plant and animal populations to be divided along Wallace's Line [62] but, to our knowledge, no prokaryotic examples have been reported. Two mutually exclusive hypotheses may explain the biogeographic separation of the Australian *B. pseudomallei *population from the more recent Asian population along Wallace's Line, both of which are reliant on the geological history of the region. Islands on the western side of Wallace's Line are part of the Eurasian tectonic plate, whereas those on the eastern side are on the Australian plate [63]. Perhaps *B. pseudomallei *was introduced into Southeast Asia after the late Miocene (approximately 12 million years ago (Ma)) collision of these two plates in the vicinity of Wallace's Line. Conversely, like other species, the biogeographic separation may have begun with the divergence of an ancestral population living in Gondwanaland. This initial divergence would be related to plate tectonic motion approximately 140 Ma when the Indian subcontinent split from Gondwanaland. Populations could have been subsequently introduced into Asia during the collision of the Indian plate and the Eurasian plate that began approximately 55 Ma [64] and then spread to the western edge of Wallace's Line. It was previously postulated that *B. pseudomallei *may have originated in Gondwanaland and dispersed with the breakup of that ancient supercontinent (the *Gondwana hypothesis*), or alternatively dispersed from Australia to Southeast Asia via the later Miocene land bridges that partially linked those regions [23]. However, low MLST allelic diversity and sharing of prevalent alleles between strains from Australia and Southeast Asia suggests that *B. pseudomallei *may actually be a much younger species [65]. A founding population must therefore have crossed Wallace's Line more recently than the late Miocene. Such an event would have to be rare to allow for genetic divergence to occur; indeed, *B. pseudomallei *does not survive well in sea water [66,67]. Although all molecular clock estimates are fraught with potential inaccuracies regarding estimates of mutation fixation rates and generation times, these two dispersion hypotheses differ by more than an order of magnitude(<12 Ma, and >140 Ma), making it likely that even a rough estimate of divergence times can discriminate between these two hypotheses. Indeed, using a range of mutation rates and generation times similar to those determined in other bacterial species, our molecular clock estimates support the hypothesis of a founding population of *B. pseudomallei *crossing Wallace's Line and becoming isolated from the larger population, with subsequent spread throughout Southeast Asia (Additional file 6). The range of our estimates for the time of divergence between the two populations (16 thousand years ago (Ka) - 225 Ka) coincides with the times of recent glacial periods when low sea levels would have maximized the potential for dispersion amongst what are now islands in the Malay Archipelago. We also dated the last common *B. pseudomallei *ancestor to between 24.9 Ka and 346 Ka and the divergence of *B. thailandensis *and *B. pseudomallei *to between 307 Ka and 4.27 Ma.

Our results demonstrate that, given large amounts of molecular data and extensive sampling, past evolutionary and biogeographic events can be reconstructed despite relatively high levels of LGT. Our use of evolutionarily informative SNPs derived from WGSs is imperative for maximizing phylogenetic resolution and reduces the likelihood that individual LGT events will corrupt the overall phylogeny, as can be expected with limited genomic sampling. Despite the problems with using limited genomic sampling schemes for determining fine scale phylogenetic patterns of relatedness in *B. pseudomallei*, such schemes are widely accessible and thus result in large data sets. Fortunately, the resolution of MLST data is sufficient for determining broad patterns of population dynamics and distribution for *B. pseudomallei *and adds this species to the growing list of bacterial species in which biogeographic structuring has been demonstrated [68,69]. More comprehensive phylogenetic and population studies will set the stage for framing and addressing further questions about single gene evolution, dispersal, and population sub-structuring.

## Methods

### SNP discovery

SNPs were detected using an in-house pipeline starting with pairwise genomic comparisons using MUMmer (Stefan Kurtz, Hamburg, Germany) [70]. We ensured orthology by requiring each SNP locus (with 100 bp on either side of the SNP) to be present in every sequenced genome and any locus that was duplicated in any genome was discarded. SNP loci with an additional SNP within 7 bp were also discarded (but see Additional file 3) to avoid possible artifacts of slight alignment errors. An average quality score of ≥15 was required for the 10 bases on each side of a SNP.

### Phylogenetic analyses

The evolutionary model that best fit the SNP data derived from whole genome comparisons was determined by Modeltest 3.6 (David Posada, Vigo, Spain) [71], analyzed under the Akaike's Information Criterion. The best fit model was used in Mr. Bayes 3.1 (John Huelsnebeck, Bret Larget, Paul van der Mark, Fredrik Ronquist, Donald Simon. Tallahassee, Florida, USA) [72] for phylogenetic inference. The Markov Chain Monte Carlo algorithm was run for 2,000,000 generations and sampled every 100 generations. A burn-in set of 2,000 trees was discarded. For all data sets, we ran the default of four chains, the log likelihood converged on a stable value well before 2,000 trees. The program SplitsTree4 (Daniel Huson and David Bryant. Tübingen, Germany) [73] was used to compute a Neighbor-Net network using uncorrected distances and equal angle splits.

### Analysis of residual homoplasies

Combining loci with different evolutionary histories, due to LGT, can result in a tree that reflects neither the history of individual genes, nor the history of the group of organisms. To determine whether LGT changed the core phylogenetic relationships among isolates, we created trees using only homoplastic SNPs. Each homoplastic SNP allele will have been inherited in a vertical manner (from mother cell to daughter cell) for the vast majority of its history, and only transferred laterally a few times. As such, the phylogenetic information content for homoplastic loci reflects both the evolutionary history of vertical descent as well as the history of LGT or convergent mutations. If LGT events involve small genomic regions and occur among a variety of lineages, the portion of phylogenetic information content due to LGT will be incongruent across genomic regions, whereas the portion that reflects the patterns of vertical descent will remain congruent. The incongruent information due to LGT will be diluted by conflicting LGT patterns from other loci, allowing the portion of phylogenetic information due to vertical descent to dictate tree structure. Thus, we identified 8,213 non-homoplastic SNPs as those loci whose allelic differences could be explained by a single change, given the phylogeny of Figure 2b. These non-homoplastic loci (Additional file 4a) were excluded from a Bayesian phylogenetic analysis of only the 6,331 remaining homoplastic SNPs as performed for Figure 2b (Additional file 4b). The differences between the tree using all 14,544 SNPs and the 6,331 homoplastic SNPs are highlighted (Additional file 4c).

### Population structure analyses

The program Structure 2.2 [40] was used to analyze 601 STs across seven loci assuming one to five populations for 10 iterations each. A burn-in of 1,000 replications was discarded and 5,000 additional replications were analyzed. The burn-in period was sufficient for stabilization of log likelihood values. The plot shown (Figure 3) for each value of *K *is based on the run with the highest likelihood value. Likewise, F_ST _values for *K *= 2 were calculated from the Structure 2.2 run with the highest likelihood value and show the divergence of each population from an estimate of ancestral allele frequencies. The level of divergence between populations (Φ_PT_) was computed with 999 permutations using GenAlEx (Rod Peakall and Peter Smouse. Canberra, Australia) [74]. For this test, STs were assigned to either an Australian or Southeast Asian population based on the geographic region designated on the MLST database.

### Calculation of other population metrics

The program START2 (Keith Jolley. Oxford, UK) [75] was used to calculate the standardized index of association [38] using only STs. Nei's [42] diversity index (D = 1-∑(allele frequency)^2^) for each MLST locus was calculated and averaged across all seven loci for each species or population. To calculate the relative contribution of recombination and mutation on allelic variation, we used the methods described elsewhere [39] except we used the program eBURST [35] to identify the most likely ancestral ST for each clonal complex. For the purposes of these calculations, the *B. pseudomallei *STs were divided into Australasian and Southeast Asian populations based on 95% assignment by the program Structure (see 'population structure analyses' above) into respective populations. The two STs assigned to *B. mallei *were excluded from these groups. The *Neisseria *MLST database contained both *N. gonorrhoeae *and *N. meningitidis *and were separated based on species labelling within the database. The *Campylobacter *database also contained both *C. jejuni *and *C. coli *STs, some of which were incorrectly labelled (EPP unpublished data). We therefore identified errors based on phylogenetic grouping and eliminated STs with ambiguous assignments. The recombination to mutation ratios for *C. jejuni *that we report here are similar to previously published values [76].

### Molecular clock calculations

Molecular clock estimates were performed by a set of in-house Perl and Java scripts. First, all protein-coding gene sequences (excluding pseudogenes) from both chromosomes of *B. pseudomallei *strain K96243 were downloaded as gene references. A BLAST-like Alignment Tool (BLAT) database [77] was constructed for all *B. pseudomallei*, *B. mallei*, and *B. thailandensis *genomes used in the comparisons and then BLAT was performed for each K96243 gene against the combined *Burkholderia *database. The BLAT output was used to align the gene sequences for all taxa relative to the coding direction in each K96243 gene. Numbers of observed synonymous (sSNPs) and potential synonymous SNP sites (sSites) were calculated for each taxon pair. Ages of divergence were calculated for each taxon pair using the following formula: Age = sSNPs/(MR × sSites × generations × 2). Divergence times at a given bifurcation were calculated by averaging the times calculated for every taxon pair that shared that bifurcation point. As mutation rates (MR) and generations per year are unknown for *B. pseudomallei*, *B. mallei*, and *B. thailandensis*, we used a range of MR [78,79] and generations per year [80] to calculate the divergence point of the Australian and Asian populations of *B. pseudomallei*.

## Abbreviations

BLAT: BLAST-like alignment tool; LGT: lateral gene transfer; MLST: multilocus sequence typing; MLVA: multiple-locus VNTR analysis; SNP: single nucleotide polymorphism; ST: sequence type; VNTR: Variable Number Tandem Repeat; WGS: whole genome sequence.

Data deposition footnote: See Additional file 1, http://bpseudomallei.mlst.net

## Authors' contributions

SHS, ZW, JC, RK, TSB and PT sequenced some of the strains. AT, MBG, ARH, RAR, MM, JEG and BJC collected isolates and epidemiological data. SBS, RA and JSBS searched for and characterized orthologous SNPs. TP and GJA performed phylogenetic analyses. TP, BL, PG and EPP performed population genetic analyses. OP, AT, PG, HH, JTF, DMW, RTO and PK contributed to interpretation and/or writing. TP wrote the manuscript.

## Supplementary Material

Additional file 1 **Supplementary Table S1**. *Burkholderia *strains with whole genome sequences. Click here for file

Additional file 2 **Supplemental Figure S1**. Bayesian cladogram of 17,718 characters shared among 38 WGSs from three species shows unresolved deep branches suggesting that this analysis can not be used to determine root placement of *B. pseudomallei/mallei *clade. Credibility values for all clades are included. Click here for file

Additional file 3 **Supplemental Figure S2**. Cladograms used to determine root placement of *B. pseudomallei/mallei *clade. Trees on left were drawn with the same 17,718 SNPs used to draw the tree in Additional file 2. Trees on right were drawn using a more inclusive set of 67,644 SNPs that were at least two nucleotides away from the nearest polymorphism. Top trees are Maximum Likelihood (ML) trees with likelihood settings from the best fit model selected by AIC in Modeltest 3.7 shown. ML trees were not bootstrapped due to the computational time required. All other trees are 50% majority-rule consensus trees with 1,000 bootstrap replicates. Middle row of trees are Maximum Parsimony trees with consistency indices labeled. The distance based Neighbor Joining algorithm was used to draw the bottom trees. Note that while most trees suggest that the first lineage to diverge from the *B. pseudomallei/mallei *clade is the Bp668 lineage, all trees suggest an Australian root to this clade. Bayesian analyses were not performed on these data as the number of SNPs exceeded the capacity of Mr. Bayes 3. Click here for file

Additional file 4 **Supplemental Figure 3**. Genomic locations of homoplastic single nucleotide polymorphisms and resulting phylogenetic tree. The genomic locations of both homoplastic and non-homoplastic SNPs are shown for each chromosome (A). Phylogenetic tree calculated using all 14,544 SNPs as in Figure 2b (B), and tree drawn using only the 6,331 homoplastic SNPs (C). Credibility values for all bifurcations are 1.00 unless otherwise noted. Topological changes resulting from only using homoplastic SNPs are indicated by a red branch. Click here for file

Additional file 5 **Supplemental Table S2**. Population metrics for recombination comparisons calculated from MLST data. Click here for file

Additional file 6 **Supplemental Figure S4**. Molecular clock estimation of the divergence of the Southeast Asian and Australasian population of *B. pseudomallei*. As neither the SNP mutation rate nor the number of generations per year are known, we assumed a range of mutation rates from 1.4 × 10^-10 ^in *E. coli *[78] to 5.2 × 10^-10 ^in *Bacillus anthracis *[79] and a range of 100 - 300 generations per year as in *E. coli *[80] to predict that the divergence of these two populations occurred between 16 Ka and 225 Ka (grey box). While actual values for *B. pseudomallei *may lie outside this range, they would have to differ greatly to support the hypothesis that the two populations diverged before the Australian and Eurasian plates collided. Using these same parameter values, we dated the last common *B. pseudomallei *ancestor to between 24.9 Ka and 346 Ka (not shown). We also dated the divergence of *B. thailandensis *and *B. pseudomallei *to between 307 Ka and 4.27 Ma. Click here for file
