# Supplementary material for: Phylogeographic reconstruction of a bacterial species with high levels of lateral gene transfer
Source: BMC Biol. 2009 Nov 18;7:78. doi: 10.1186/1741-7007-7-78 (PMC2784454; doi:10.1186/1741-7007-7-78)
Supplement: Additional file 1 — Supplementary Table S1. Burkholderia strains with whole genome sequences. [file 1741-7007-7-78-S1.DOC]

**Supplemental Table 1. *Burkholderia* strains with whole genome sequences.**

| **Strains** | **Species** | **Status** | **Refseq/Source** | **Strain info** |  |
| --- | --- | --- | --- | --- | --- |
| 1026b | *B. pseudomallei* | shotgun | Dr. R. Kaul | 1993 Northeast Thailand |  |
| 406e | *B. pseudomallei* | shotgun | NZ_AAMM00000000 | 1988 Northeast Thailand |  |
| 112 | *B. pseudomallei* | shotgun | NZ_ABBP00000000 | 1992 Northeast Thailand |  |
| Pasteur52237 | *B. pseudomallei* | shotgun | NZ_AAHV00000000 | 1964 Vietnam |  |
| 1710a | *B. pseudomallei* | shotgun | NZ_AAHS00000000 | 1996 Northeast Thailand (see also 1710b) |  |
| 1710b | *B. pseudomallei* | Complete | NC_007434,NC_007435 | 1999 Northeast Thailand relapse of same patient with 1710a |  |
| 14 | *B. pseudomallei* | shotgun | NZ_ABBJ00000000 | 1990 Phillipines |  |
| 9 | *B. pseudomallei* | shotgun | NZ_ABBL00000000 | 1988 Pakistan |  |
| S13 | *B. pseudomallei* | shotgun | NZ_AAHW00000000 | Singapore |  |
| 1106a | *B. pseudomallei* | Complete | NC_009076,NC_009078 | 1993 Northeast Thailand (see also 1106b) |  |
| 1106b | *B. pseudomallei* | shotgun | NZ_AAMB00000000 | 1996 Northeast Thailand relapse of same patient with 1106a |  |
| 22 | *B. pseudomallei* | Complete | Dr. P. Tan | 1996 Singapore |  |
| B7210 | *B. pseudomallei* | shotgun | NZ_ABBN00000000 | 1970 Malaysia* |  |
| 91 | *B. pseudomallei* | shotgun | NZ_ABBK00000000 | Southeast Asia* |  |
| K96243 | *B. pseudomallei* | Complete | NC_006350,NC_006351 | 1996 Northeast Thailand |  |
| 7894 | *B. pseudomallei* | shotgun | NZ_ABBO00000000 | 1962 Ecuador |  |
| BCC215 | *B. pseudomallei* | shotgun | NZ_ABBR00000000 | Brazil |  |
| MSHR1655 | *B. pseudomallei* | shotgun | NZ_AAHR00000000 | 2003 Northern Australia |  |
| DM98 | *B. pseudomallei* | shotgun | NZ_ABBI00000000 | Australia |  |
| MSHR668 | *B. pseudomallei* | Complete | NC_009074,NC_009075 | 1995 Northern Australia |  |
| NCTC13177 | *B. pseudomallei* | shotgun | NZ_ABBQ00000000 | Western Australia |  |
| MSHR305 | *B. pseudomallei* | shotgun | NZ_AAYX00000000 | 1994 Northern Australia |  |
| E208** | *B. pseudomallei* | shotgun | Dr. T. Brettin | 1990 Ecuador |  |
| TXDOH | *B. thailandensis* | shotgun | NZ_ABBD00000000 | 2003 USA |  |
| E254 | *B. thailandensis* | shotgun | Dr. T. Brettin | Northeast Thailand |  |
| 4 | *B. thailandensis* | shotgun | NZ_ABBH00000000 | Northern Australia |  |
| ATCC700388 | *B. thailandensis* | shotgun | NZ_AACX00000000 | Central Thailand. Derived from same isolate as E264 |  |
| E264 | *B. thailandensis* | Complete | NC_007651,NC_007650 | Central Thailand. Derived from same isolate as ATCC700388 |  |
| MSMB43 | *B.* sp | shotgun | NZ_ABBM00000000 | Australia |  |
| ATCC10399 | *B. mallei* | shotgun | NZ_AAHN00000000 | 1942 Southern China |  |
| FMH | *B. mallei* | shotgun | NZ_AAIQ00000000 | 2000 Human passaged derivative of ATCC 23344 from the same infection as JHU |  |
| GB horse4 | *B. mallei* | shotgun | NZ_AAHO00000000 | A horse passaged derivative of ATCC 23344 |  |
| ATCC23344 | *B. mallei* | Complete | NC_006348,NC_006349 | 1944 Burma |  |
| JHU | *B. mallei* | shotgun | NZ_AAIR00000000 | 2000 Human passaged derivative of ATCC 23344 from the same infection as FMH |  |
| PRL-20 | *B. mallei* | shotgun | NZ_AAZP00000000 | 2005 Pakistan |  |
| SAVP1 | *B. mallei* | Complete | NC_008785,NC_008784 | Unknown |  |
| 2002721280 | *B. mallei* | shotgun | NZ_AANX00000000 | 1952 Iran |  |
| NCTC10229 | *B. mallei* | Complete | NC_008836,NC_008835 | 1961 Hungary |  |
| NCTC10247 | *B. mallei* | Complete | NC_009080,NC_009079 | 1960 Turkey |  |
| C6786 | *B. oklahomensis* | shotgun | NZ_ABBG00000000 | 1973 USA |  |
| EO147 | *B. oklahomensis* | shotgun | NZ_ABBF00000000 | 1977 USA |  |
| BdAUO158 | *B. dolosa* | shotgun | NZ_AAKY00000000 |  |  |
| Bu | *B. ubonensis* | shotgun | NZ_ABBE00000000 |  |  |
| *Information reflects our attempts to determine isolate origins due to past incomplete record keeping. | | | | | |

** While in press, we discovered that this isolate is probably from Australia rather than Ecuador.
