# Supplementary figures and images for: Phylogeographic reconstruction of a bacterial species with high levels of lateral gene transfer
Source: BMC Biol. 2009 Nov 18;7:78. doi: 10.1186/1741-7007-7-78 (PMC2784454; doi:10.1186/1741-7007-7-78)

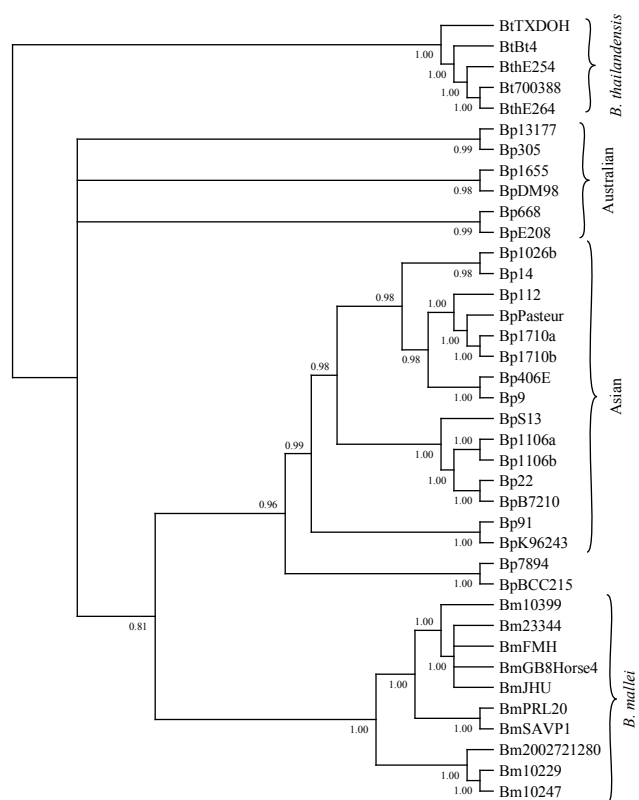

Supplement: Additional file 2 — Supplemental Figure S1. Bayesian cladogram of 17,718 characters shared among 38 WGSs from three species shows unresolved deep branches suggesting that this analysis can not be used to determine root placement of B. pseudomallei/mallei clade. Credibility values for all clades are included. [file 1741-7007-7-78-S2.PDF]

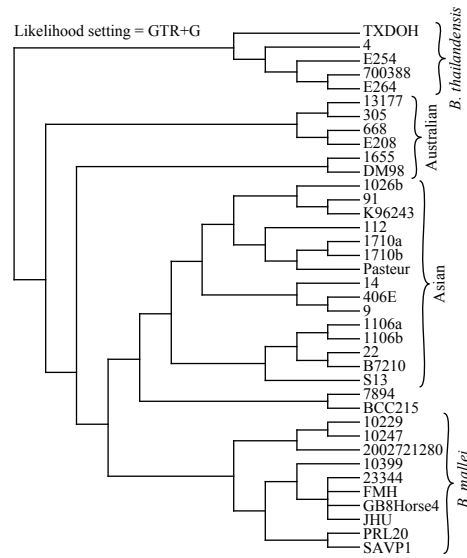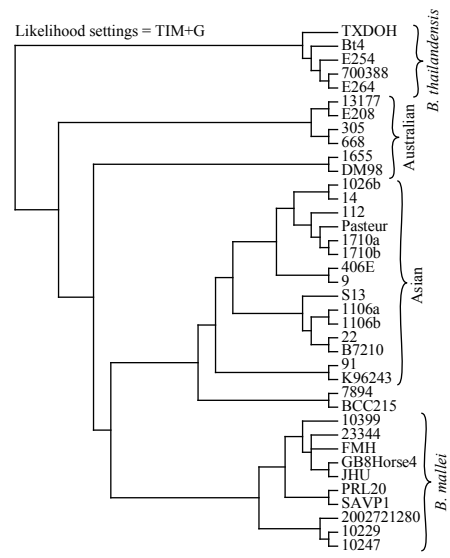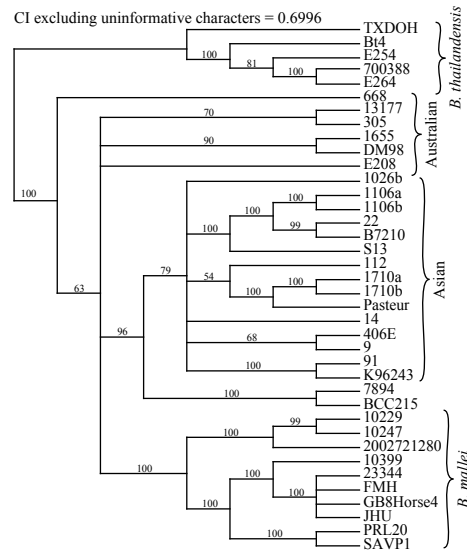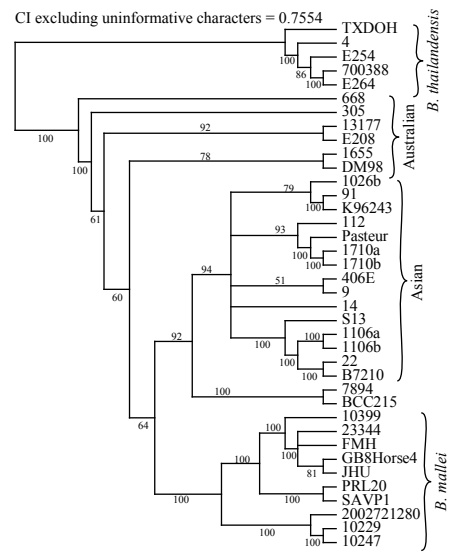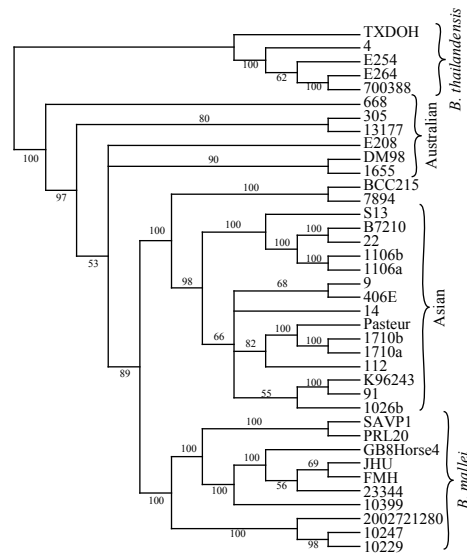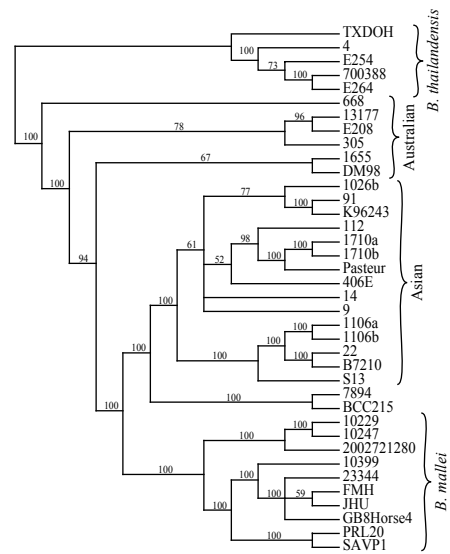

Supplement: Additional file 3 — Supplemental Figure S2. Cladograms used to determine root placement of B. pseudomallei/mallei clade. Trees on left were drawn with the same 17,718 SNPs used to draw the tree in Additional file 2. Trees on right were drawn using a more inclusive set of 67,644 SNPs that were at least two nucleotides away from the nearest polymorphism. Top trees are Maximum Likelihood (ML) trees with likelihood settings from the best fit model selected by AIC in Modeltest 3.7 shown. ML trees were not bootstrapped due to the computational time required. All other trees are 50% majority-rule consensus trees with 1,000 bootstrap replicates. Middle row of trees are Maximum Parsimony trees with consistency indices labeled. The distance based Neighbor Joining algorithm was used to draw the bottom trees. Note that while most trees suggest that the first lineage to diverge from the B. pseudomallei/mallei clade is the Bp668 lineage, all trees suggest an Australian root to this clade. Bayesian analyses were not performed on these data as the number of SNPs exceeded the capacity of Mr. Bayes 3. [file 1741-7007-7-78-S3.PDF]

A.

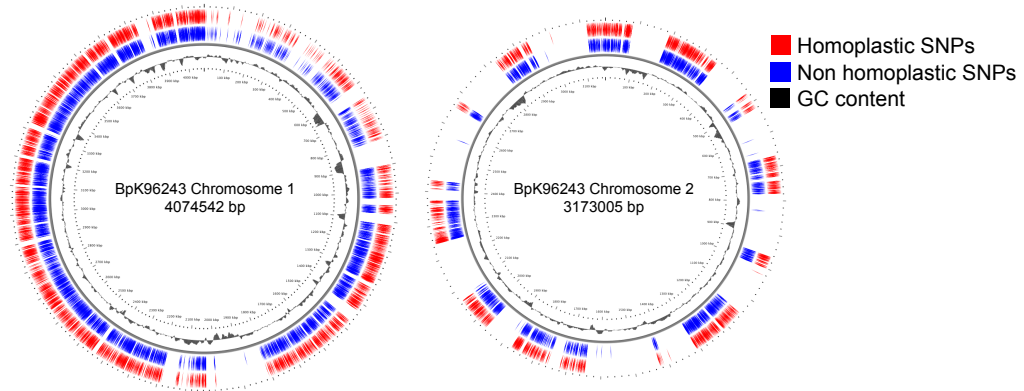

B.

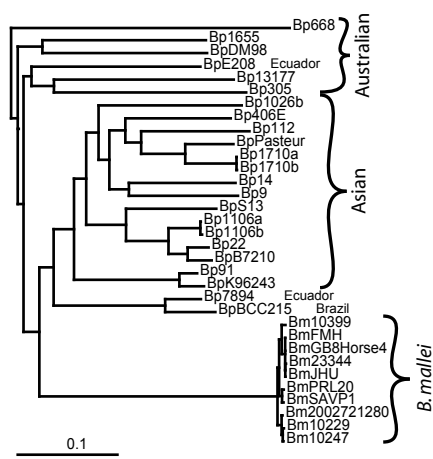

C.

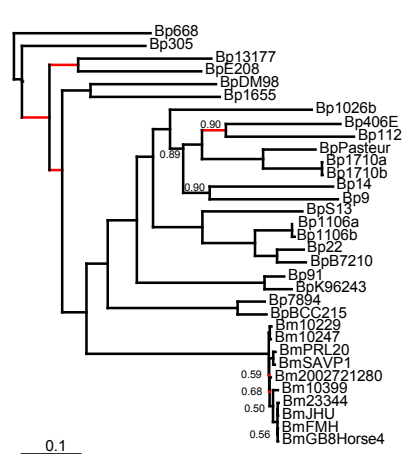

Supplement: Additional file 4 — Supplemental Figure 3. Genomic locations of homoplastic single nucleotide polymorphisms and resulting phylogenetic tree. The genomic locations of both homoplastic and non-homoplastic SNPs are shown for each chromosome (A). Phylogenetic tree calculated using all 14,544 SNPs as in Figure 2b (B), and tree drawn using only the 6,331 homoplastic SNPs (C). Credibility values for all bifurcations are 1.00 unless otherwise noted. Topological changes resulting from only using homoplastic SNPs are indicated by a red branch. [file 1741-7007-7-78-S4.PDF]

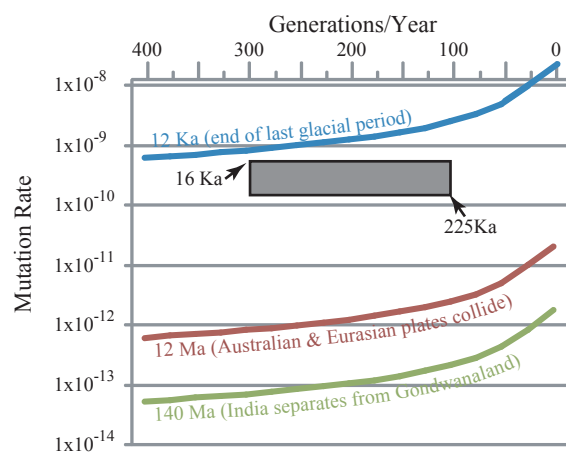

Supplement: Additional file 6 — Supplemental Figure S4. Molecular clock estimation of the divergence of the Southeast Asian and Australasian population of B. pseudomallei. As neither the SNP mutation rate nor the number of generations per year are known, we assumed a range of mutation rates from 1.4 × 10^-10^ in E. coli [78] to 5.2 × 10^-10^ in Bacillus anthracis [79] and a range of 100 - 300 generations per year as in E. coli [80] to predict that the divergence of these two populations occurred between 16 Ka and 225 Ka (grey box). While actual values for B. pseudomallei may lie outside this range, they would have to differ greatly to support the hypothesis that the two populations diverged before the Australian and Eurasian plates collided. Using these same parameter values, we dated the last common B. pseudomallei ancestor to between 24.9 Ka and 346 Ka (not shown). We also dated the divergence of B. thailandensis and B. pseudomallei to between 307 Ka and 4.27 Ma. [file 1741-7007-7-78-S6.PDF]
