# Supplementary material for: Phylogeographic reconstruction of a bacterial species with high levels of lateral gene transfer
Source: BMC Biol. 2009 Nov 18;7:78. doi: 10.1186/1741-7007-7-78 (PMC2784454; doi:10.1186/1741-7007-7-78)
Supplement: Additional file 5 — Supplemental Table S2. Population metrics for recombination comparisons calculated from MLST data. [file 1741-7007-7-78-S5.DOC]

**Supplemental Table 2. Population metrics for recombination comparisons calculated from MLST data.**

|  | # STs | I_a_^s^ | r/m allele | r/m site | site/allele | Diversity |
| --- | --- | --- | --- | --- | --- | --- |
| *H. pylori* | 1931 | 0.292 | 1.5 | 17 | 11.333333 | 0.9990517 |
| *N. meningitidis* | 6944 | 0.134 | 4.75 | 100 | 21.052632 | 0.9199952 |
| *S. pneumoniae* | 4242 | 0.0345 | 8.9 | 60 | 6.741573 | 0.8903728 |
| *N. gonorrhoeae* | 116 | -0.0016 | 5.7 | 9 | 1.5789474 | 0.5277518 |
| *S. pyogenes* | 468 | 0.0408 | 2.35 | 7.02 | 2.987234 | 0.8858077 |
| *B. pseudomallei* | 599 | 0.0332 | 18.67 | 29.33 | 1.5714286 | 0.7089082 |
| *Aust Bp* | 267 | 0.0066 | 18 | 33 | 1.8333333 | 0.6972324 |
| *SEA Bp* | 266 | 0.0181 | 30 | 44 | 1.4666667 | 0.6285222 |
| *C. jejuni* | 2378 | 0.153 | 5.97 | 50.47 | 5.875 | 0.9134014 |
| *E. faecium* | 472 | 0.1267 | 5 | 24 | 4.8 | 0.7173662 |
| *S. epidermidis* | 211 | 0.1327 | 2.5 | 10 | 4 | 0.7001832 |
| *S. aureus* | 1319 | 0.1969 | 0.067 |  | 0 | 0.8739992 |
